# Analysis of organochlorine pesticides residues in fish from Edko Lake (North of Egypt) using eco-friendly method and their health implications for humans

**DOI:** 10.1007/s43188-020-00085-8

**Published:** 2021-03-08

**Authors:** Moustafa A. Abbassy, Moustafa A. Khalifa, Atef M. K. Nassar, Eman E. Nour El-Deen, Yehia M. Salim

**Affiliations:** 1 Faculty of Agriculture, Plant Protection Department, Damanhour University, PO Box 59, Damnhour, Elbehera Egypt; 2 Faculty of Agriculture, Department of Pesticides Chemistry and Toxicology, Kafrelsheikh University, Kafrelsheikh, Egypt

**Keywords:** Organochlorine pesticides (OCPs), GC-ECD, GC-ITD, Edko lake, QuEChERS

## Abstract

Levels of organochlorine pesticides (OCPs) residues in tilapia fish and their health risk associated with the consumption of fish from Edko lake, Egypt were determined. The quick, easy, cheap, effective, rugged, and safe method (QuEChERS) was used for extraction and clean-up of 18 OCPs residues from fish followed by GC-ECD and GC-ITD analytical tools were employed for identification of OPs. Out of the 18 OCPs, only heptachlorepoxide, p,p-DDE, dieldrin, p,p-DDD, and endrin ketone were detected in muscles of fish with concentrations of 0.1144, 0.2119, 0.4352, 0.1196, and 0.1323 µg/Kg bw, respectively. Results of the risk assessment calculations showed that dieldrin had a health risk index of more than one. This means that there was a health risk to adults associated with the consumption of fish. On the other hand, heptachlorepoxide, p,p-DDE, p,p-DDD, and endrin ketone did not show any direct health risk. Contamination of fish, collected from Edko lake in Egypt, with residues of OCPs might pose health implications if fish were consumed fresh.

**Supplementary Information:**

The online version contains supplementary material available at 10.1007/s43188-020-00085-8.

## Introduction

The indiscriminate and irresponsible use of pesticides in agriculture causes environmental problems especially to aquatic system by altering the quality of water and affecting the physiological and biochemical characteristics of non-target fish [1–3]. Once in the aquatic environment, pesticides are absorbed by aquatic organisms and concentrated in the trophic food chain thus endangering the life of fish and other organisms [4–9]. It has been found that greater than 80% of the total intake of pesticides residues by humans is through the food chain via consumption of contaminated food [10]. Fish accumulate contaminants directly from water and/or through the food chain. In case of OCPs, they could persist and be accumulated in living organisms especially in aquatic organisms [11].

Generally, the ability of fish to metabolize the OCPs is moderate, therefore, contamination load in fish relies on the degree of pollution in the surrounding environment [12]. Fish absorb OCPs directly from water or by ingesting contaminated food. The region of accumulation of pesticides within fish varies with the route of uptake [13]. Moreover, the amounts of OCPs in gills reflect their concentration in water, while the liver store all pesticides. Accordingly, fish could be considered as one of the significant bioindicators of pesticides pollution in freshwater systems [14, 15]. Therefore, comprehensive, yet precise, analytical methods of these group of pesticides are needed.

Exhaustive extraction methods are usually used for the determination of pesticides in water and food samples including soaking with organic solvents, Soxhlet, and supercritical fluid extraction [16]. However, these methods suffer several challenges including cost, simplicity, and rapidity since a relatively large amount of matrix is required, solvent consumption is high, and further time-consuming cleanup steps might be required. Alternatively, the quick, easy, cheap, effective, rugged, and safe (QuEChERS) method is the most readily used technique for the extraction of pesticides residues [17]. The present study was achieved on fish samples from Edko lake that is a major fish production site in Egypt. Therefore, the objectives of current study were to a) determine OCPs residues in fish samples of Nile tilapia and b) assess the potential health hazard of detected OCPs residue levels.

## Materials and methods

### Pesticide standards and chemicals

A mixture of certified reference standard pesticides containing 18 organochlorine pesticides (OCPs), at a concentration of 2000 µg (± 0.5%)/ml for each pesticide, was obtained from SUPLECO company (Bellefonte, PA, USA). The OCP mixture composed of α-HCH (99.7%), γ-HCH (99.9%), β-HCH (98.9%), heptachlor (99.9%), Δ-HCH (99.5%), aldrin (98.9%), heptachlorepoxide (99.9%), endosulfan I (99.9%), p,p-DDE (99.2%), dieldrin (99.2%), endrin (96.9%), p, p-DDD (96.1%), endosulfan II (99.9%), p, p-DDT (98.9%), endrin aldehyde (98.4%), endosulfan sulfate (99.4%), methoxychlor (99.9%), and endrin ketone (99.5%). A standard solution of 500 ng/ml was prepared by dissolving the OCPs mixture in methanol and stored at − 4 °C. Working standard solutions were prepared by proper dilution from the stock solution.

Ethyl acetate, methylene chloride, methanol, and acetonitrile were of HPLC-analysis grade (Merck, Darmstadt, Germany). Water was purified using Millipore-Q Water Purification System (Millipore, Bedford, MA, USA). Primary secondary amine (PSA), sodium sulfate, and magnesium sulfate were purchased from reputed chemical suppliers.

### Study areas and samples collection

Fish samples were collected from Edko Lake and control fish were reared in the laboratory. The collected fish samples were transferred immediately to the laboratory in an icebox and then dissected to separate muscle, liver, and gills. Then dissected samples were freeze-dried at − 60 °C, ground using mini-chopper, and stored at -20ºC until the analysis for pesticide residues was performed.

### Extraction and cleanup of OCPs residues from fish samples

The OCPs residues were extracted from liver, gills, and muscles of fish samples using the quick, easy, cheap, effective, rugged, and safe (QuEChERS) method [17]. Approximately, 2 g of lyophilized fish samples (liver, gills, and muscles) were placed in a 50 ml falcon tube. About 15 ml of acetonitrile acidified with 1% of glacial acetic acid were added and shaken vigorously for 30 s. Then, 6 g of magnesium sulfate and 1.5 g of sodium chloride were added, and the tubes were shaken again for 1 min. The tubes were centrifuged at 4000 rpm for 4 min at 4ºC (Hermle Labortechnik GmbH, Siemensstr D-78564 Wehingen, Germany). Two ml from the supernatant were transferred to 15 ml falcon tubes and cleaned-up with 0.3 g of MgSO_4_, 0.1 g of PSA, 0.1 g of C_18_, and 0.015 g of activated charcoal. Then the tubes were shaken for 30 s and centrifuged at 4000 rpm for 4 min at 4ºC. The supernatant was transferred to auto-sampler vials for the GC analysis.

### Instrumentation for OCPs analysis in extracts of fish samples

Extracts (1–2 µl) were analyzed for the presence of 18 OCPs utilizing a GC-ECD (Varian, 3400, Walnut, Greek, CA, USA) equipped with a Varian 8200 autosampler. Chromatographic separations of the OCPs residues were achieved using HP-608 fused silica capillary column (30 m × 0.53 mm i.d 0.5 µm film thickness). Helium was used as the carrier gas and nitrogen as the makeup gas. Separation conditions for GC-ECD were as the following: initial column temperature was 80° C for 6 min, increased to 215 °C at a rate of 15 °C/min (hold for 1 min), then to 230 °C at 5 °C/min, and finally to 290 °C at 5 °C/min (hold for 2 min). The GC-ECD was controlled by a computer system, which has EI-MS libraries (Willey spectral library).

### Validation of OCPs analysis in extracts of fish samples using the mass spectrometry

Identification and confirmation for the presence of target compounds were accomplished using a benchtop Gas Chromatography-Ion Trap Detector, (GC-ITD), which consists of a Varian 3800 GC interfaced to a Saturn 2000, which was operated in splitless mode (purge time set at 1 min) and maintained at a temperature of 250 °C. The chromatographic separation was achieved using an HP-5MS capillary column (30 m × 250 µm and 0.25 µm film thickness). The carrier gas was helium at a constant flow rate of 1.1 ml/min. The separation temperature program was initially set at 85 °C for 0.3 min, increased to 150 °C (hold for 4 min) at a rate of 30 °C/min, then to 185 °C at a rate of 2 °C/min, and finally to 290 °C (hold for 5 min) at a rate of 4 °C/min. The ITD was operated in electron impact ionization mode (EI) at 70 eV and temperature at 220 °C. EI spectra were monitored by scanning ions within the range of 50–500 amu. The target compounds were identified by their full scan mass spectra and retention time using the total ion current as a monitor to give a total ion chromatogram (ITC). The use of the full scan mode allows comparing the spectrum obtained for interested compounds with the EI-MS libraries. Besides, selective ion monitoring (SIM) mode was used for the identification and confirmation of tested compounds according to their selective specific ions for the compound of interest (Table 1).

**Table 1:** Chromatographic data obtained from the analysis of multi-standards of 18 OCPs injected through GC-ECD that was operated under optimized conditions and obtained ions from the GC-ITD that were used for confirmation, identification, and quality control (limit of detection (LOD) and limit of quantification (LOQ); µg/Kg) of the investigated compounds in fish samples

OCs	Ions (m/z)	tR^a^	*R*^b^	*T*^c^	LOQ	LOD
α-HCH	183, 219, 111	13.31 ± 1.1	2.3	1.01 ± 0.025	0.056	0.013
γ-HCH	181, 109, 219	14.30 ± 1.2	2.1	1.02 ± 0.021	0.056	0.015
β-HCH	181, 109, 219	15.20 ± 0.9	2.2	1.01 ± 0.025	0.044	0.017
Heptachlor	100, 272, 237	15.37 ± 1.3	2.1	1.02 ± 0.036	0.073	0.019
δ-HCH	109, 183, 219	16.40 ± 1.4	2.2	1.01 ± 0.015	0.055	0.018
Aldrin	66, 79, 263	17.47 ± 1.1	2.2	1.03 ± 0.025	0.042	0.009
Heptachlorepoxide	81, 253, 263	18.61 ± 1.2	2.5	1.02 ± 0.010	0.076	0.019
Endosulfan Ι	241, 195, 339	19.10 ± 1.1	2.4	1.02 ± 0.012	0.044	0.017
p,p-DDE	246, 176, 318	19.8 ± 0.8	2.3	1.04 ± 0.015	0.038	0.009
Dieldrin	79, 263, 277	20.80 ± 1.1	1.5	1.02 ± 0.015	0.062	0.018
Endrin	81, 263, 67	20.70 ± 1.1	1.5	1.03 ± 0.025	0.069	0.018
p,p- DDD	235, 165, 199	21.47 ± 1.3	1.6	1.04 ± 0.015	0.079	0.019
Endosulfan Π	195, 207, 241	21.85 ± 1.1	1.8	1.01 ± 0.021	0.081	0.021
p,p-DDT	235, 199, 165	22.32 ± 1.2	1.5	1.01 ± 0.015	0.068	0.018
Endrin aldehyde	67, 345, 250	22.72 ± 1.1	1.5	1.02 ± 0.012	0.064	0.018
Endosulfan sulfate	387, 272, 237	23.08 ± 1.5	1.5	1.02 ± 0.015	0.078	0.019
Methoxychlor	227, 308, 238	24.50 ± 1.2	2.5	1.01 ± 0.021	0.082	0.023
Endrin ketone	67, 139, 317	25.22 ± 1.1	2.3	1.02 ± 0.026	0.081	0.022

^a^Reproducibility of tR for each analyte was evaluated during 2 months with minimum of 10 injection of reference standard mixture solution and RSD (%) were determined

^b^Resolution was determined and the acceptable limit, *R* not less than 1.5

^c^tailing factor (*T*) was determined according to USP [22] and acceptable limit (*T* range from 0.9 to 1.1)

### OCPs quantification, linearity of GC-ECD response, and calibration

The concentrations of OCPs were determined from 5 points (0.01–10 µg/L) calibration curve of each tested compound. The integrated peak areas were plotted versus the concentration. To check the linearity of the calibration graphs, the concentrations of each compound were run 15 times and correlation coefficient (R^2^) for each compound was calculated.

### OCPs detection limit (LOD) and limit of quantification (LOQ) on GC–ITD-SIM

The LOD and LOQ were determined according to PAM [18] and EPA [19]. The LOD was calculated as the lowest concentration of OCP which provided a chromatographic peak height 3 times greater than the average baseline noise (at the same retention time). The LOQ was determined as the corresponding value of 10 times the baseline noise in the chromatogram of the blank sample.

### Method precision

For the assessment of precision of the proposed method, repeatability (intra-day assay precision) and intermediate precision (inter-day assay precision) were determined. The intra-day and inter-day precision were determined by repeating the analysis of five fortified fish samples on the same day and 5 consecutive days, respectively. The average percentage of recovery for each compound and the relative standard deviation (RSD) were calculated.

### Method selectivity

The selectivity of the proposed analytical procedure was determined by assessing the separation pattern of reference standards mixture of fortified and non-fortified fish samples (muscle, gill, and liver) (Supplementary information). Additionally, the GC–ITD-SIM technique was very selective in the detection of analytes in investigated samples where the corresponding ions related to each analyte was mentioned in Table 1.

### Extraction efficiency (Recovery tests)

The efficiency of QuEChERS extraction method for the targeted 18 OCPs pesticides from fish samples was examined by calculating the average percentage of recoveries (R%) from fortified blank fish samples. Also, the percent relative standard deviation (RSD%) was estimated (Table 2). Exactly, laboratory fish samples (liver, gills, and muscle) were fortified with the OCPs mixture. Fortified fish samples were extracted and analyzed as previously mentioned. Average percentages of recoveries (R%) were determined and RSD % for recoveries were calculated. All data of residue analysis were corrected according to these obtained recovery percentages values.

**Table 2:** Average recovery percentages ± relative standard deviation (RSD) for 18 OCPs extracted from fortified laboratory blank-liver, -gills, and –muscles tissue samples of fish using QuEChERS technique with two levels of the multi-standards mixture (0.1 and 1 µg/Kg)

OCPs	0.1 µg/Kg			1 µg/Kg		
	Liver	Gills	Muscles	Liver	Gills	Muscles
α-HCH	93.1 ± 1.3	95.1 ± 3.2	91.2 ± 6.1	94.1 ± 1.3	94.1 ± 3.2	90.2 ± 6.1
γ-HCH	95.1 ± 1.3	96.1 ± 4.2	94.2 ± 5.1	93.1 ± 1.3	94.1 ± 4.2	93.2 ± 5.1
β-HCH	96.2 ± 2.3	97.3 ± 3.2	96.1 ± 6.1	97.2 ± 2.3	99.3 ± 3.2	95.1 ± 6.1
Heptachlor	97.3 ± 4.1	98.1 ± 2.2	97.1 ± 6.1	98.3 ± 4.1	96.1 ± 2.2	98.1 ± 6.1
δ-HCH	98.3 ± 5.1	99.1 ± 3.1	99.1 ± 5.1	96.3 ± 5.1	97.1 ± 3.1	97.1 ± 5.1
Aldrin	93.3 ± 2.2	98.2 ± 2.2	100.1 ± 3.2	92.3 ± 2.2	95.2 ± 2.2	98.1 ± 3.2
Heptachlorepoxide	99.1 ± 3.2	99.2 ± 3.2	99.1 ± 5.2	97.1 ± 2.2	98.2 ± 3.2	99.1 ± 5.2
Endosulfan Ι	98.1 ± 2.5	100.1 ± 1.2	100.1 ± 3.2	98.1 ± 2.5	98.1 ± 1.2	97.1 ± 3.2
p,p-DDE	100.1 ± 3.5	92.2 ± 3.2	99.1 ± 6.1	98.1 ± 3.5	95.2 ± 3.2	98.1 ± 6.1
Dieldrin	98.2 ± 2.5	99.1 ± 3.2	94.2 ± 3.2	96.2 ± 3.5	98.1 ± 3.2	93.2 ± 3.2
Endrin	93.2 ± 5.1	100.2 ± 3.4	99.2 ± 4.1	93.2 ± 5.1	98.2 ± 3.4	98.2 ± 4.1
p,p-DDD	94.5 ± 3.2	93.2 ± 2.3	95.3 ± 5.2	95.5 ± 3.2	95.2 ± 2.3	93.3 ± 5.2
Endosulfan Π	95.6 ± 2.2	94.2 ± 2.4	91.1 ± 3.2	96.6 ± 2.2	96.2 ± 2.4	96.1 ± 3.2
p,p-DDT	96.7 ± 3.2	98.2 ± 3.1	99.1 ± 3.3	97.7 ± 3.2	95.2 ± 3.1	98.1 ± 3.3
Endrin aldehyde	99.1 ± 3.1	99.1 ± 4.1	100.3 ± 2.3	98.1 ± 3.1	96.1 ± 4.1	97.3 ± 2.3
Endosulfan sulphate	98.1 ± 2.5	98.2 ± 3.2	92.1 ± 4.1	96.1 ± 1.5	95.2 ± 3.2	96.1 ± 4.1
Methoxychlor	99.9 ± 2.3	100.1 ± 2.2	91.2 ± 3.2	97.9 ± 1.3	98.1 ± 2.2	95.2 ± 3.2
Endrin ketone	98.9 ± 2.5	95.6 ± 3.2	93.2 ± 3.2	96.9 ± 2.5	94.6 ± 3.2	97.2 ± 3.2

*n* = 3 Replicates

### Health risk assessment

Health risk assessment of consumers from the intake of pesticides contaminated fish was expressed as health risk index (HI). The HI was obtained by dividing the estimated daily intake (EDI) by their corresponded values of acceptable daily intake (ADI) as reported by WHO/FAO [20] as shown by the equation:HI = EDI/ADI.

Then, the estimation of daily intake (EDI) of any pesticide was determined using the equation: EDI = C*D/B.

where C, D, and B represent the concentration of pesticide residues in fish (µg/g bw), average daily intake of fish estimated at 46.027 g/person/day for adults, and average body weight considered to be 70 kg for adults, respectively. When HI is less than 1, the food is considered acceptable. If the HI was greater than 1, the food is considered a hazard to consumers [21–23].

### Statistical analysis

Data were statistically analyzed as a completely randomized design (CRD) at a significance level of *p* ≤ 0.05. All statistical analyses were performed using SAS 9.3 (SAS Institute Inc., Cary, NC, USA). Data were expressed as means ± RSD.

## Results

### GC-ECD and GC–MS conditions and optimization

The chromatographic conditions were optimized through several trials to achieve high sensitivity, high resolution, and symmetrical peak shapes (no tailing) for separation and determination of OCPs using GC-ECD (Supplementary information). Results in Table 1 revealed that OCPs were separated from each other at the baseline with resolution values (R) ranged between 1.5 and 2.5. The separated peaks on GC-ECD chromatogram had symmetrical shapes with tailing factor (*T*) between 1.01 and 1.04 (acceptable range is from 0.9 to 1.1 [22]. Also, the RSD% values for retention time (tR) for OCPs were less than 1.5, which indicated the stability of the chromatographic system. GC–MS (GC-ITD) was used for further confirmation of identified OCPs in extracts of fish samples through comparing the 3 major ions in the MS spectrum of OCPs in samples with that of the reference standard (Table 1).

### Method validation

The accuracy of the employed analytical method was determined via the calculation of average percentages recoveries for OCPs and the percents relative standard deviation (RSD %) of recoveries from fortified blank samples of fish (Table 2). The average percentages of recoveries and the RSD% of recoveries at 0.1 µg/Kg level of OCPs standards ranged from 93.1 ± 1.3 to 100.1 ± 3.5%, 92.2 ± 3.2 to 100.2 ± 3.4%, and 91.1 ± 3.2 to 100.3 ± 2.3% from fortified liver, gill, and muscle samples, respectively. At 1 µg/Kg level of the reference material, the recovery percentages ranged from 92.3 ± 2.2 to 98.3 ± 4.1%, 94.1 ± 3.2 to 99.3 ± 3.2%, and 90.2 ± 6.1 to 99.1 ± 5.2% from liver, gills, and muscle samples, respectively (Table 2). Results of residue analysis in fish samples were corrected according to obtained recovery percentage values. Moreover, data in Table 2 showed that applied method is accurate and met the acceptable criteria of ICH [24] where recoveries were from 70 to 130% and RSD% values were below 20%.

### Linearity, limits of detection (LOD), limits of quantification (LOQ), precision, and selectivity of the analytical method

Five points (0.01–10 µg/Kg) calibration curves of the tested OCPs were constructed. The integrated peak areas were plotted versus the concentration. In order to check for the linearity of calibration graphs, the correlation of coefficient (R^2^) for each compound was calculated. The calibration data of the targeted 18 OCPs showed good linearity for the response of ECD detector where *R*^*2*^ values were > 0.99997.

The LOD and LOQ were determined according to PAM [18] and EPA [19] using fortified blank fish samples with 18 OCPs standards mixtures. The LOD was calculated as the lowest concentration of OCPs which provided a chromatographic peak height of three times the average baseline of noise (at the same retention time).The LOQ was determined corresponding a value of 10 times the noise peak at the same retention time. The LOD and LOQ of 18 OCPs in fish samples using the proposed method were found to be in the range 0.01–0.02 µg/Kg wet weight and 0.04–0.08 µg/Kg wet weight, respectively (Table 1).

For the assessment of the precision of proposed method, repeatability (intra-day-assay precision) and intermediate precision (inter-day assay precision) were determined. According to the data in Table 3, it was clear that the developed method was precise as the RSD% values were less than 20% as recommended by ICH [24] guidelines.

**Table 3:** Inter–day and intra-day precision data obtained from analysis of a multi-standards of 18 OCPs in extracts of fortified laboratory blank-liver, -gills, and -muscles tissue of fish samples (0.1 µg/Kg bw) and extracted using QuEChERS technique and analyzed using GC-ECD

OCPs	Inter-day precision (R% ± RSD)			Intra-day precision (R% ± RSD)		
	Liver	Gills	Muscles	Liver	Gills	Muscles
α-HCH	97.1 ± 4.1	94.1 ± 4.1	96.1 ± 4.1	98.1 ± 4.1	95.1 ± 5.1	99.1 ± 5.1
γ-HCH	92.2 ± 5.1	93.2 ± 5.1	92.2 ± 5.1	90.2 ± 6.2	94.2 ± 7.2	90.2 ± 7.2
β-HCH	95.2 ± 6.1	94.2 ± 6.1	92.2 ± 6.1	96.1 ± 5.2	95.1 ± 7.2	98.1 ± 7.2
Heptachlor	94.2 ± 5.1	98.2 ± 5.1	97.2 ± 5.1	92.2 ± 7.1	93.2 ± 7.1	94.2 ± 7.1
δ-HCH	95.6 ± 3.0	95.8 ± 3.0	95.8 ± 3.0	97.2 ± 6.1	98.2 ± 5.1	96.2 ± 5.1
Aldrin	95.9 ± 3.9	98.9 ± 3.9	97.9 ± 3.9	95.9 ± 5.1	95.9 ± 5.1	92.9 ± 41
Heptachlorepoxide	93.9 ± 4.1	95.9 ± 4.1	93.9 ± 4.1	92.6 ± 5.3	95.9 ± 6.3	93.9 ± 6.3
Endosulfan Ι	96.1 ± 5.1	96.1 ± 5.1	98.1 ± 5.1	97.1 ± 4.5	97.1 ± 4.5	97.1 ± 4.5
p,p-DDE	94.2 ± 3.2	94.2 ± 3.2	94.2 ± 3.2	95.2 ± 6.2	95.2 ± 6.2	95.2 ± 6.2
Dieldrin	97.1 ± 4.2	96.1 ± 2.2	95.1 ± 2.2	95.8 ± 5.1	96.8 ± 5.1	96.8 ± 5.1
Endrin	97.1 ± 6.5	95.1 ± 6.5	96.1 ± 6.5	98.2 ± 7.3	96.2 ± 7.3	99.2 ± 7.3
p,p-DDD	91.1 ± 4.1	91.1 ± 4.1	94.1 ± 4.1	91.3 ± 5.1	92.3 ± 61	92.3 ± 61
Endosulfan Π	98.1 ± 6.5	98.1 ± 6.5	95.1 ± 6.5	98.2 ± 6.7	99.2 ± 6.7	97.2 ± 6.7
p,p-DDT	92.1 ± 4.1	93.1 ± 4.1	94.1 ± 4.1	90.3 ± 7.1	92.3 ± 7.1	95.3 ± 7.1
Endrin aldehyde	94.2 ± 5.1	94.2 ± 5.1	96.2 ± 5.1	95.2 ± 6.1	95.2 ± 6.1	97.2 ± 6.1
Endosulfan sulfate	92.2 ± 4.1	93.2 ± 4.1	90.2 ± 4.1	90.8 ± 5.6	94.8 ± 5.6	90.8 ± 5.6
Methoxychlor	91.3 ± 5.1	95.3 ± 5.1	91.3 ± 5.1	92.2 ± 4.5	93.2 ± 3.5	90.2 ± 4.5
Endrin ketone	93.2 ± 3.4	91.2 ± 3.4	93.2 ± 3.4	93.3 ± 4.2	92.3 ± 4.2	92.3 ± 4.2

The selectivity of the proposed analytical procedures was clear where no interfering peaks from the endogenous materials of fish constituents were observed at the retention times of all analytes. Data in Table 1, about resolution values, showed that 18 OCPs were adequately resolved from each other and from fish constituents. Additionally, the SIM technique provided a very selective detection tool for analytes in investigated fish samples. All these validated data of the developed method for analysis of 18 OCPs in extracts of fish were deemed acceptable according to ICH [24].

### Residues levels of OCPs in fish samples collected from Lake Edko

The developed and validated analytical method was applied for residue analysis of 18 OCPs in fish samples collected from investigated area, Edko lake. For the determination of OC pesticide residues in fish samples, QuEChERS technique was used because of its greater simplicity. This method covers a wide pesticide range (polar, pH-dependent compounds), simple (no laborious steps with, minimal sources of errors), cheap, solvent consumption is minimum (10 mL acetonitrile, GC- and LC-amenable), and practically no glassware is needed [17, 25, 26].

The mean concentrations of OCPs residues in tissues (liver, gills, and muscles) of Nile Tilapia (*Oreochromis niloticus* L., 1758) from Edko lake were presented in Table 4. The detected compounds were heptachlorepoxide, dieldrin, p,p-DDE, p,p-DDD, and endrin ketone. Concentrations of these pesticides were 0.2245 ± 0.0254, 1.4189 ± 0.0435, 0.9742 ± 0.0751, 1.4039 ± 0.0266, and 0.1382 ± 0.0325 µg/Kg in liver tissue, respectively. In gills tissue, the concentrations of the same pesticides were 0.1243 ± 0.0254, 1.4208 ± 0.0287, 0.7198 ± 0.0324, 1.2983 ± 0.0284, and 0.2371 ± 0.0263 µg/Kg, respectively. Also, the same OCPs were detected in muscles of fish at 0.1144 ± 0.0324, 0.2119 ± 0.0267, 0.4352 ± 0.0383, 0.1196 ± 0.0429, and 0.1323 ± 0.0270 µg/Kg, respectively (Table 4).

**Table 4:** OCs residue levels (µg/Kg ± SD) in liver, gills, and muscles of blank (B) and fish samples collected from Lake Edko (L), El-Behera Governorate and extracted using QuEChERS technique and analyzed using GC-ECD

OCPs	Liver		Gills		Muscles	
	B	L	B	L	B	L
Heptachlorepoxide	ND	0.2254 ± 0.0254	ND	0.1243 ± 0.0254	ND	0.1144 ± 0.0324
p,p-DDE	ND	1.4189 ± 0.0435	ND	1.4208 ± 0.0287	ND	0.2119 ± 0.0267
Dieldrin	ND	0.9742 ± 0.0751	ND	0.7198 ± 0.0324	0.02 ± 0.0163	0.4352 ± 0.0383
p,p-DDD	ND	1.4039 ± 0.0266	ND	1.2983 ± 0.0284	ND	0.1196 ± 0.0429
Endrin Ketone	ND	0.1382 ± 0.0325	ND	0.2371 ± 0.0263	ND	0.1323 ± 0.0270

### Health risk assessment of consumption of fish contaminated with OCPs

Out of the analyzed 18 OCPs, p,p-DDE, p, p-DDD, dieldrin, heptachlorepoxide, and endrin ketone were detected in the liver, gills, and muscles of Tilapia fish samples. As shown in Table 5, hazard indices of 0.069, 0.039, 2.893, 0.145, and 0.427 were computed for adults consuming fish contaminated with p,p-DDE, p,p-DDD, dieldrin, heptachlorepoxide, and endrin ketone, respectively. The estimated hazard indices of p,p-DDD, p,p-DDE, heptachlorepoxide, and endrin ketone were < 1, which highlight no hazard to human. While the HI of dieldrin exceeded one, which pose a direct hazard to human health.

**Table 5:** Mean concentrations of detected residues of OCPs and effective daily intake (EDI) and the health index (HI) values that are associated with the consumption of muscles of fish from Edko Lake

OCPs	Mean (mg/Kg)	ADI* (mg/Kg bw/ day)	EDI	HI
p,p-DDE	0.00021	0.001	0.00014	0.138
p,p-DDD	0.00012	0.001	0.00008	0.079
Dieldrin	0.00044	0.0001	0.00029	2.893
Heptachlorepoxide	0.00011	0.0001	0.00007	0.723
Endrin ketone	0.00013	0.0002	0.00009	0.427

*ADI Acceptable daily intake (Codex Alimentarius EU website [27] and Australian Government Department of Health [20])

## Discussion

From data reported herein, it was clear that GC-ECD analytical method that was validated with GC-ITD was simple and rapid analytical tool (chromatographic run time about 30 min) for the separation, identifications, confirmation, and quantifications of the 18 OCPs under investigation. Moreover, the applied method was accurate and met the acceptable criteria of ICH, where recoveries were from 70 to 130% and RSD% values were below 20%. It was clear that the developed method was precise as the RSD% values were less than 20% as recommended by ICH [24] guidelines. The selectivity of the proposed analytical procedures was clear where no interfering peaks from the endogenous materials of fish constituents were observed at the retention times of all analytes. Also, the 18 OCPs were adequately resolved from each other and from fish constituents. Additionally, the SIM technique provided a very selective detection tool for analytes in investigated fish samples. All these validated data of the developed method for analysis of 18 OCPs in extracts of fish were deemed acceptable according to ICH [24].

The present results showed that liver and gill had the highest levels of p,p-DDT in the form of p,p-DDE and p,p-DDD metabolites, although the use of DDT has been banned in Egypt. This may be due to the persistence of these compounds in the environment and their bioaccumulation in the aquatic organisms. Also, the residue levels of p,p-DDE and dieldrin in muscle samples and p,p-DDE, dieldrin, and p, p-DDD in both liver and gills tissues exceeded the permissible maximum residue limit, MRL [27]; 1 µg/Kg for DDE and DDD and 0.2 µg/Kg for Dieldrin. Previous studies reported similar results about OCP residues in liver of fish samples [28, 29] and [30] and in gills [31] and [32]. This may be due to the high lipophilic and hydrophobic nature of these metabolites, and their possibility retention in the organic phase of sediment and organisms. Also, OCP residues were found in tilapia fish samples (*Tilapia zilli*) from Lake Bosomtwi [22]. They indicated that DDE was the predominant residue in analyzed samples with concentrations ranged from 4.10 to 7.25 ng/g tissue in 58% of fish samples. However, DDT was detected in 78% of fish samples at concentrations ranged from 3.40 to 4.65 ng/g tissue. Aldrin was the lowest one (in 16% of fish samples) at concentrations ranged from 0.3 to 0.49 ng/g tissue. Also, the levels of OCPs were determined in selected edible fish, red belly tilapia and catfish from Volta, Bosomtwi, and Weija Lakes in Ghana during 2008. The highest amounts were attributed to DDT metabolites followed by chlordane, HCH, and lindane along with small amounts of heptachlor, octachlor, and oxychlordane. Yet the detected amounts of DDT in tissue samples were lower than US Food and Drug Administration (FDA) tolerance limit [33].

Dieldrin was detected in all the fish samples, while aldrin was not be detected because aldrin was converted to dieldrin in fish organs. The results obtained were consistent with those of the United States Department of Health & Human Services (USDHHS) [34], which showed that aldrin is rapidly converted into dieldrin in plant and animal tissues. In addition, it is bio-concentrated in lipids such as animal fats and plant waxes because of its nonpolar nature. Dieldrin is one of the most persistent pesticides and is not readily metabolized in water, similar to DDT, and its metabolites and has restricted digestion and excretion from the body [35]. It is, however, easily absorbed and transported throughout the blood of vertebrates and hemolymph of invertebrates. The difference in patterns of these contaminants in muscles, gills and liver may reflect difference in contaminant metabolism, content and composition of lipids, or the degree of blood perfusion in the various tissues [36].

Health risks associated with OC metabolites are well documented [37]. Obtained results of health risk assessment showed that dieldrin in fish of Edko lake recorded health risk index more than one and this means that there was health risk associated with adult consumption of this fish. Current results of dieldrin pose a great potential for chronic toxicity to human after the consumption of contaminated fish. Similarly, Ezemonye et al. [38] reported elevated risk quotient of dieldrin in *Tilapia zilli* and *Clarias gariepinus* fish species. Accumulation of this persistent pesticides in body tissues as a result of consumption of contaminated fish might lead to acute or chronic health effects. Also, it would be advisable to assess their potential health effects against human, and non-target organisms that got exposed (directly or indirectly) to these group of banned pesticides yet they found in the environment. Moreover, accumulation pattens and amounts should be further investigated. Other monitored insecticides, heptachlorepoxide, p,p-DDE, p,p-DDD and endrin ketone, did not show any direct health risk although their presence in fish of Edko lake.

## Supplementary Information

Below is the link to the electronic supplementary material.

Supplementary file 1 (DOCX 517 KB)

## Abbreviations

ADI: Acceptable daily intake
EDI: Estimated daily intake
EI-MS: Electron impact ionization mode
EPA: Environmental protection agency
FAO: Food and agriculture organization
GC-ECD: Gas chromatography with electron capture detector
GC-ITD: Gas chromatography with ion trap mass spectrometry detector
HI: Health risk index
HPLC: High performance liquid chromatography
ICH: The International Council for Harmonisation of Technical Requirements for Pharmaceuticals for Human Use
ITC: Total ion chromatogram
LOD: Limit of detection
LOQ: Limit of quantification
MgSO_4_: Magnesium Sulfate
OCPs: Organochlorine pesticides
PAM: Pesticide analytical manual
PSA: Primary secondary amine
QuEChERS: Quick, easy, cheap, effective, rugged, and safe method
RSD%: Percent relative standard deviation
R%: Average percentage of recoveries
SIM: Selective ion monitoring ion
WHO: World Health Organization
