# Supplementary material for: Analysis of organochlorine pesticides residues in fish from Edko Lake (North of Egypt) using eco-friendly method and their health implications for humans
Source: Toxicol Res. 2021 Mar 8;37(4):495–503. doi: 10.1007/s43188-020-00085-8 (PMC8476673; doi:10.1007/s43188-020-00085-8)
Supplement: Supplementary file 1 — (DOCX 517 KB) [file 43188_2020_85_MOESM1_ESM.docx]

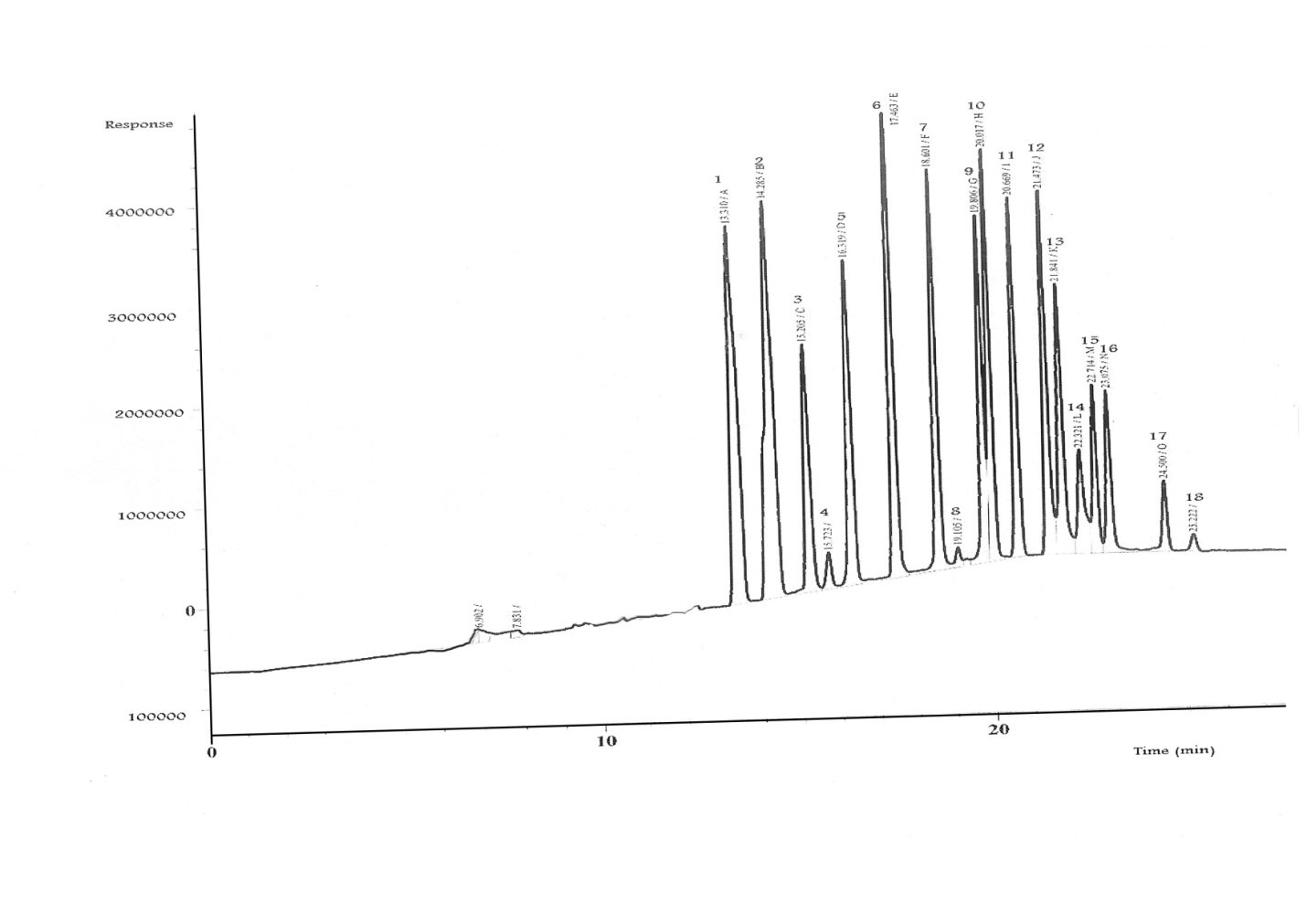


Appendix 1. GC-ECD chromatogram of separation patterns of the multi-reference standards of 18 OCPs at 0.1 µg /L dissolved in methanol: 1) α–HCH, 2) γ-HCH, 3) β–HCH, 4) Heptachlor, 5) δ-HCH, 6) Aldrin, 7) Heptachlorepoxide, 8) Endosulfan Ι, 9) p,p-DDE, 10) Dieldrin, 11) Endrin, 12) p,p-DDD, 13) Endosulfan Π, 14) p,p-DDT, 15) Endrin aldehyde, 16) Endosulfan sulfate, 17) Methoxychlor, and 18) Endrin Ketone.


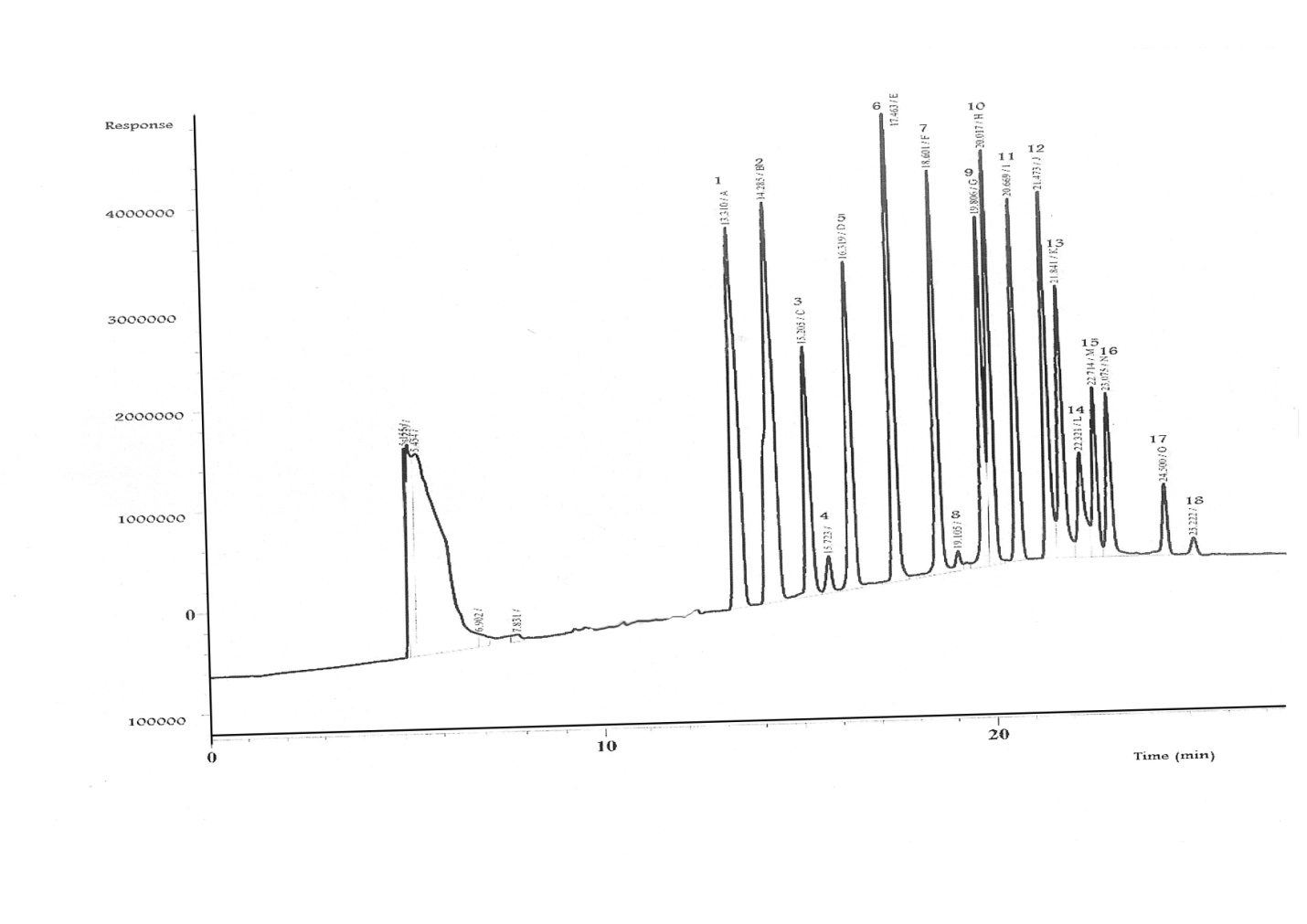


Appendix 2. GC-ECD chromatogram of separation patterns of the QuEChERS extract of blank muscle sample of laboratory reared-fish fortified with the multi-reference standards of 18 OCPs at 0.1 µg /kg b.w.: 1) α–HCH, 2) γ-HCH, 3) β–HCH, 4) Heptachlor, 5) δ-HCH, 6) Aldrin, 7) Heptachlorepoxide, 8) Endosulfan Ι, 9) p,p-DDE, 10) Dieldrin, 11) Endrin, 12) p,p-DDD, 13) Endosulfan Π, 14) p,p-DDT, 15) Endrin aldehyde, 16) Endosulfan sulfate, 17) Methoxychlor, and 18) Endrin Ketone.


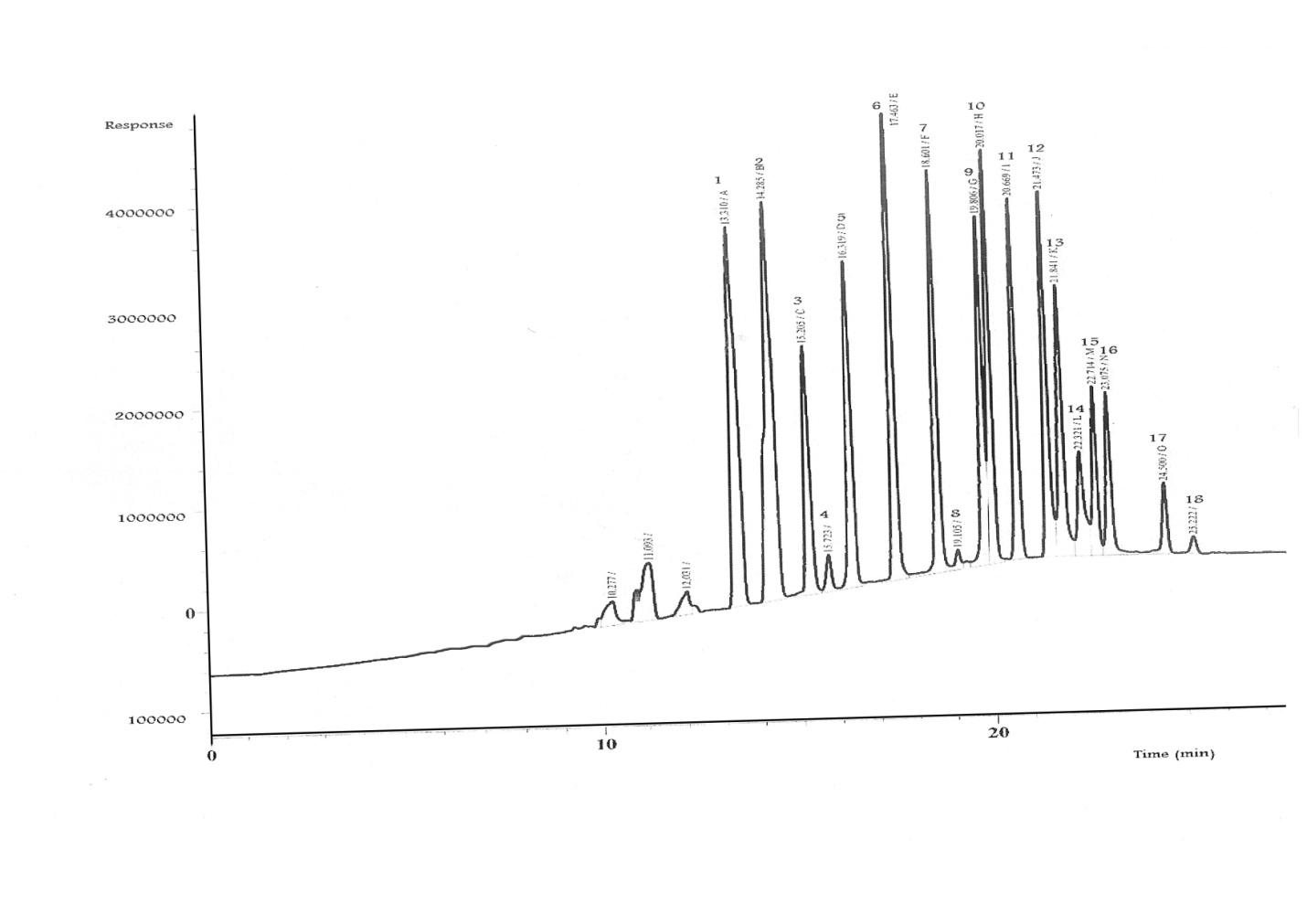


Appendix 3. GC-ECD chromatogram of separation patterns of the QuEChERS extract of blank gills sample of laboratory reared-fish fortified with the multi-reference standards of 18 OCPs at 0.1 µg /kg b.w.: 1) α–HCH, 2) γ-HCH, 3) β–HCH, 4) Heptachlor, 5) δ-HCH, 6) Aldrin, 7) Heptachlorepoxide, 8) Endosulfan Ι, 9) p,p-DDE, 10) Dieldrin, 11) Endrin, 12) p,p-DDD, 13) Endosulfan Π, 14) p,p-DDT, 15) Endrin aldehyde, 16) Endosulfan sulfate, 17) Methoxychlor, and 18) Endrin Ketone.


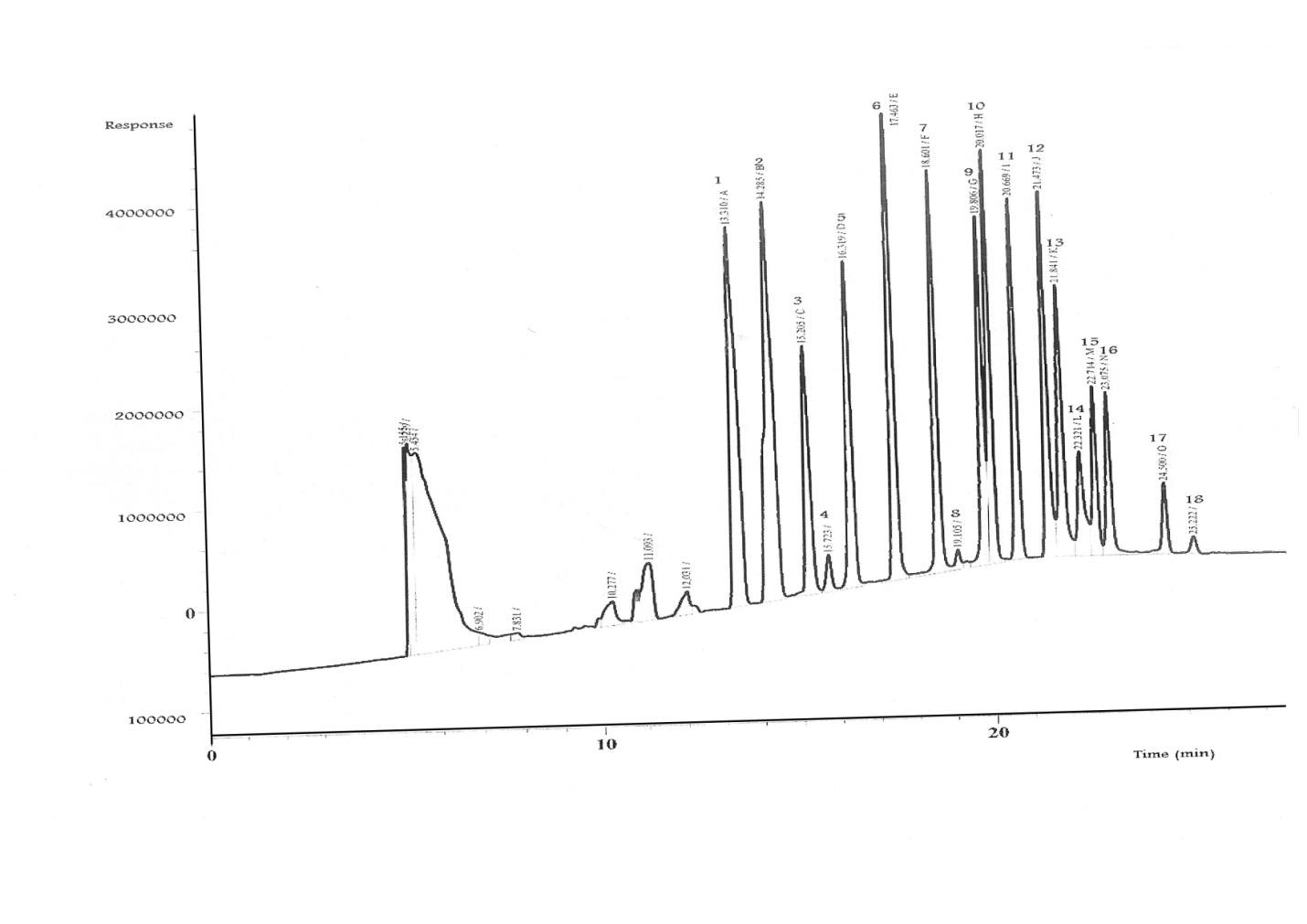


Appendix 4. GC-ECD chromatogram of separation patterns of the QuEChERS extract of blank liver sample of laboratory reared-fish fortified with the multi-reference standards of 18 OCPs at 0.1 µg /kg b.w.: 1) α–HCH, 2) γ-HCH, 3) β–HCH, 4) Heptachlor, 5) δ-HCH, 6) Aldrin, 7) Heptachlorepoxide, 8) Endosulfan Ι, 9) p,p-DDE, 10) Dieldrin, 11) Endrin, 12) p,p-DDD, 13) Endosulfan Π, 14) p,p-DDT, 15) Endrin aldehyde, 16) Endosulfan sulfate, 17) Methoxychlor, and 18) Endrin Ketone.
